# Eye care during the COVID-19 public health emergency: a WHO perspective

**Published:** 2020-09-01

**Authors:** Silvio Mariotti, Stuart Keel, Alarcos Cieza

**Affiliations:** 1 Medical Officer: Vision and Eye Care Programme, Department of Noncommunicable Diseases, World Health Organization, Geneva, Switzerland.; 2 Technical Officer: Vision and Eye Care Programme, Department of Noncommunicable Diseases, World Health Organization, Geneva, Switzerland.; 3 Coordinator: Blindness and Deafness Prevention, Disability and Rehabilitation, Department of Noncommunicable Diseases, World Health Organization, Geneva, Switzerland.

**During the COVID-19 pandemic, the World Health Organization emphasises care for people with disabilities and non-urgent eye conditions, safety, documenting innovations and further integration of eye health in health systems.**

**Figure:**
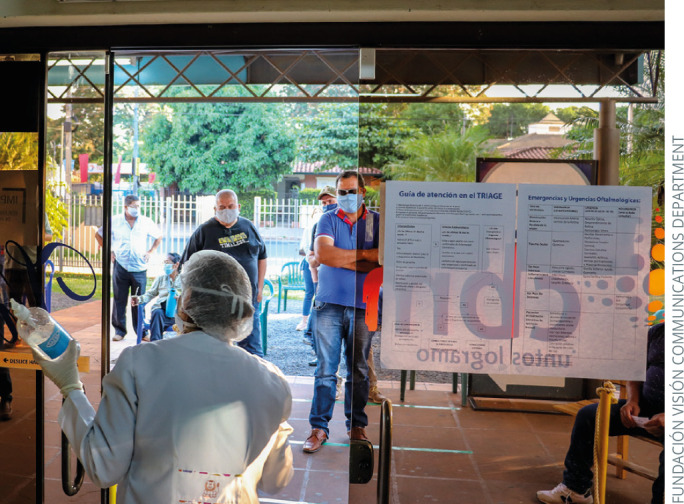
Patients wait their turn outside Fundación Visión Eye Hospital. **PARAGUAY**

The World Health Organization (WHO) declared the COVID-19 outbreak a Public Health Emergency of International Concern on 30 January 2020. The outbreak – now a global pandemic – has affected the lives of all segments of the population around the world, including health care workers. The purpose of this article is to emphasise some important points, from a WHO perspective, for health workers involved in eye care.

## Take measures to protect and care for people with disabilities

People with one or more disabilities are particularly vulnerable during the COVID-19 pandemic.

People with disabilities may be disproportionately affected by the outbreak. This could be due to disruptions to the services and support they rely on, difficulties they may experience in implementing basic hygiene measures and enacting social distancing and barriers in accessing public health services and information. In some cases, pre-existing health conditions can leave them at a higher risk of permanent, serious illness if they contract the virus.

Barriers experienced by people with disabilities can be reduced if key stakeholders take appropriate action. WHO guidance on appropriate actions and measures that governments, service providers, communities and people with disabilities themselves can take to protect individuals with a disability during the COVID-19 pandemic is available at **https://bit.ly/Cov19disability**

## Use personal protective equipment from approved sources only

There are current disruptions in the global supply chain of PPE due to the surge in demand that has been driven by COVID-19. Every country has registered sources of certified PPE for patients and providers. WHO recommends to only use PPE derived from approved sources, and to avoid self-made, or social media-advertised, distributors of PPE of uncertain quality. Further information on the rational use of PPE for COVID-19 can be found at **https://bit.ly/CovPPE**

## Ensure patients who need non-urgent eye care are not left behind

While the focus during the pandemic is to maintain the delivery of essential eye care services and to avoid interruptions in access to medicines for patients with chronic eye conditions, patients requiring non-urgent care need not feel left behind.

Take advantage of telehealth and other technological advances not only to practice effective triage and facilitate coordination between care providers, but also to ensure that patients needing non-urgent appointments remain engaged and informed, adhere to their prescribed treatment strategies and preventive actions, and continue in their care-seeking behaviours as we emerge from the pandemic.

## Document and share innovative approaches and their impact

The COVID-19 response has shown us that innovative workforce management approaches, such as temporary relocation, task-sharing and role delegation, can be effective to address acute workforce shortages or inefficiencies. It is important that those working in eye care document any new or innovative approaches that have been adopted during the pandemic, as well as their impact on eye care delivery and outcomes. This can provide the basis for planning high quality health services and implementation research (following the pandemic) to verify if and how these approaches can be scaled up to improve clinical care and people’s lives, and to enhance equity in the provision of high-quality eye care service delivery.

## Integrate eye care within health systems

The most significant recommendation of the World Report on Vision is to ensure that eye care is integrated within the health sector service delivery system and its strategic plans. This recommendation was relevant before the pandemic, as there are currently at least 1 billion people with vision impairment that could have been prevented or is yet to be addressed, and vast inequities exist in access to eye care services between and within countries.

This is relevant during the pandemic given that, if eye care is an integral part of the health sector, there is an increased likelihood that it will play a more prominent role within the response plan of the health sector. Integration will be vital when implementing eye care programmes after the COVID-19 pandemic to ensure their sustainability and provide continuity of care for patients.

